# Diagnostic Ability of Manual Calcification Length Assessment on Non-Electrocardiographically Gated Computed Tomography for Estimating the Presence of Coronary Artery Disease

**DOI:** 10.3390/jcm13082255

**Published:** 2024-04-12

**Authors:** Ryota Watanabe, Yuichi Saito, Satoshi Tokimasa, Hiroyuki Takaoka, Hideki Kitahara, Masato Yamanouchi, Yoshio Kobayashi

**Affiliations:** 1 Department of Cardiovascular Medicine, Chiba University Graduate School of Medicine, Chiba 260-8670, Chiba, Japan; navefuta@gmail.com (R.W.); tapy21century@yahoo.co.jp (H.T.); hidekita.0306@gmail.com (H.K.); yuiryosuke@msn.com (Y.K.); 2 Department of Cardiology, Chiba Rosai Hospital, Ichihara 290-0003, Chiba, Japan; yfd02270@nifty.com (S.T.); masato_yamanouti@chibah.johas.go.jp (M.Y.)

**Keywords:** coronary artery disease, coronary calcification, computed tomography, electrocardiographic gating

## Abstract

**Background:** Coronary artery calcification score (CACS) on electrocardiography (ECG)-gated computed tomography (CT) is used for risk stratification of atherosclerotic cardiovascular disease, which requires dedicated analytic software. In this study, we evaluated the diagnostic ability of manual calcification length assessment on non-ECG-gated CT for epicardial coronary artery disease (CAD). **Methods:** A total of 100 patients undergoing both non-ECG-gated plain CT scans with a slice interval of 1.25 mm and invasive coronary angiography were retrospectively included. We manually measured the length of the longest calcified lesions of coronary arteries on each branch. The relationship between the number of coronary arteries with the length of coronary calcium > 5, 10, or 15 mm and the presence of epicardial CAD on invasive angiography was evaluated. Standard CACS was also evaluated using established software. **Results:** Of 100 patients, 49 (49.0%) had significant epicardial CAD on angiography. The median standard CACS was 346 [7, 1965]. In both manual calcium assessment and standard CACS, the increase in calcium burden was progressively associated with the presence of epicardial CAD on angiography. The receiver operating characteristic curve analysis showed similar diagnostic abilities of the two diagnostic methods. The best cut-off values for CAD were 2, 1, and 1 for the number of vessels with calcium > 5, 10, and 15 mm, respectively. Overall, the diagnostic ability of manual calcium assessment was similar to that of standard CACS > 400. **Conclusions:** Manual assessment of coronary calcium length on non-ECG-gated plain CT provided similar diagnostic ability for the presence of significant epicardial CAD on invasive angiography, as compared to standard CACS.

## 1. Introduction

Ischemic heart disease, an atherosclerotic cardiovascular disease (ASCVD), is the leading cause of morbidity and mortality worldwide. Vascular calcification is a feature of atherosclerosis associated with adverse cardiovascular outcomes. In patients with cardiovascular risk factors, vascular calcification and advanced atherosclerosis are related to cardiovascular mortality. The amount of coronary artery calcification (CAC) on computed tomography (CT) represents the development of atherosclerosis and is a strong predictor of cardiovascular events [1,2]. The detection of CAC on CT has been proposed as a method to enhance traditional risk stratification, and previous studies have shown that CAC can improve risk prediction as compared to that of conventional risk factor-based algorithms [2]. For instance, a previous study with very long-term follow-up (median of 14.6 years) showed that the higher CAC score (CACS) was associated with increased all-cause mortality in a stepwise manner, with 15-year mortality of 3.5% in patients with zero CACS and 18.0% in those with CACS ≥ 400 [3]. The absence of CAC on CT scans is associated with a very low ASCVD risk and, thus, is commonly used to rule out coronary artery disease (CAD). In the US guidelines, among adults at intermediate risk (10-year risk of ASCVD ≥ 7.5% to <20%) or selected adults at borderline risk (5% to <7.5% 10-year risk), measuring CACS is recommended for the guidance of clinician–patient risk discussion [4]. In such patients, the guidelines indicate that if the CACS is zero, it is reasonable to withhold statin therapy and reassess in 5 to 10 years, while when CACS is 1 to 99, the initiation of statin therapy may be reasonable in patients ≥ 55 years of age [4]. When CACS is ≥100, the statin therapy is recommended [4]. Although specific recommendations vary widely, international guidelines endorse the use of CACS to estimate the risks of ASCVD [4,5,6]. The potential candidates for CACS assessment who may benefit from recognizing their CACS is 0 for the primary prevention of CAD are suggested as follows: (1) Patients reluctant to initiate statin (and other lipid-lowering) therapy who wish to precisely understand their risk and potential for benefit, (2) patients concerned about the need to reinstitute statin therapy after discontinuation for statin-associated symptoms, (3) older patients (men, 55–80 years; women, 60–80 years) with low burden of risk factors who question whether they would benefit from statin therapy, and (4) middle-aged adults (40–55 years) with pooled cohort equation-calculated 10-year risk of ASCVD [7].

From a technical perspective, to minimize motion artifacts and optimize scoring, CACS is usually evaluated on electrocardiography (ECG)-gated CT [8], while non-ECG-gated CT scans for CACS assessment have been also described in previous reports, in which a strong correlation in CACS between CT scans with and without ECG-gated acquisition techniques was found [9]. Although the standard acquisition protocol includes axial multidetector CT performed with prospective ECG-gating, non-ECG-gated CT may be useful in the CAC assessment. Indeed, a meta-analysis including five studies with 1316 individuals for assessing the diagnostic agreement of CACS between ECG-gated and non-ECG-gated CT scans showed an excellent correlation [9]. In addition, the prognostic performance of non-ECG-gated CT was also validated [9]. Nonetheless, standard CACS analysis requires dedicated equipment and software irrespective of ECG-gating [8]. Thus, it would be clinically useful if CAC on non-ECG-gated CT images could be assessed without specific techniques and equipment. In the present study, we assessed the diagnostic ability of simple manual evaluation methods of CAC on non-ECG-gated CT for predicting the presence of epicardia CAD.

## 2. Materials and Methods

### 2.1. Study Design

This was a retrospective, single-center study. From April 2019 to April 2022, a total of 175 patients underwent non-ECG-gated plain CT scans for various reasons and invasive coronary angiography within a 2-year interval at Chiba Rosai Hospital. Patients with acute coronary syndrome (*n* = 63) and previous coronary stent implantation (*n* = 12) were excluded. Thus, a total of 100 patients were included in the present study. Epicardial CAD was defined as the presence of coronary diameter stenosis > 50% on invasive coronary angiography. Cardiovascular risk factors such as hypertension, diabetes, dyslipidemia, and current smoking were defined according to the Japanese Association of Cardiovascular Intervention and Therapeutics criteria. A blood test was performed on admission. Hypertension was defined as a previous diagnosis of hypertension or previous antihypertensive medications, or newly diagnosed hypertension during hospitalization with systolic blood pressure ≥ 140 mm Hg and/or diastolic blood pressure ≥ 90 mm Hg. Diabetes was defined as a previous diagnosis of diabetes or previous glucose-lowering medications or a level of hemoglobin A1c ≥ 6.5%. Dyslipidemia was defined as having low-density lipoprotein cholesterol ≥ 140 mg/dL, high-density lipoprotein cholesterol < 40 mg/dL, or fasting triglycerides > 150 mg/dL, or a previous diagnosis of dyslipidemia. Low- and high-density lipoprotein cholesterol levels were evaluated in either a fasting or non-fasting state. Other blood test findings including hemoglobin and creatinine were also evaluated. In addition, patients with a history of smoking within the past year were defined as being current smokers. Chronic kidney disease was defined as an estimated glomerular filtration rate < 60 mL/min/1.73 m^2^. Indications of invasive coronary angiography such as heart failure, suspected angina, preoperative testing, brady arrhythmia, the presence of peripheral artery disease, and vasospastic angina were also evaluated. The present study was performed in accordance with the Declaration of Helsinki. This study was approved by the Ethical Committee of Chiba Rosai Hospital (approval number: 12-05), and informed consent for the present study was obtained in an opt-out manner.

### 2.2. Computed Tomography Analysis

**Figure 1:**
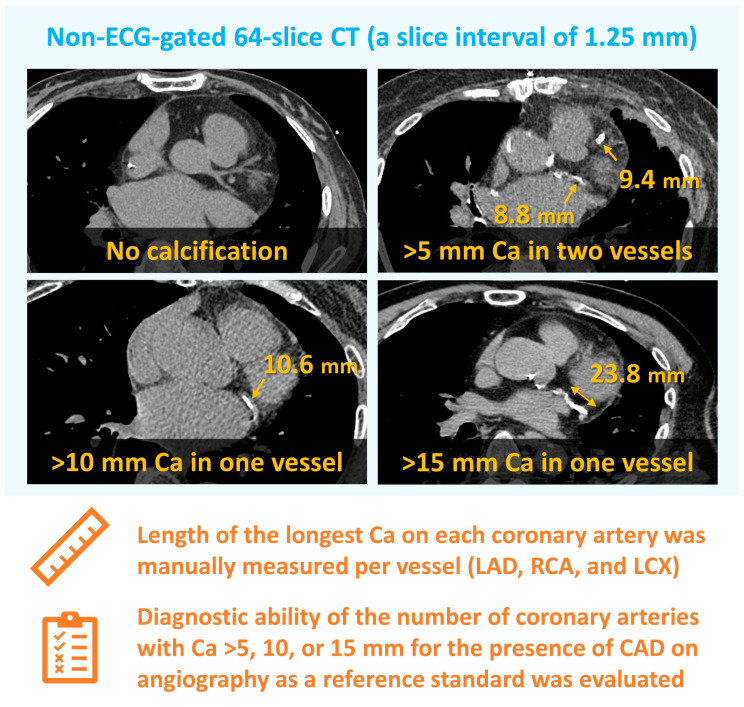
Methods of manual calcium assessment of coronary arteries. Ca, calcium; CAD, coronary artery disease; CT, computed tomography; ECG, electrocardiography; LAD, left anterior descending coronary artery; LCX, left circumflex artery; RCA, right coronary artery.

Plain CT scans were performed using 64-slice CT (Discovery CT750HD; GE Healthcare, Milwaukee, WI, USA) with a slice interval of 1.25 mm at a tube voltage of 120 kV without ECG gating. We evaluated CAC with two different approaches, a manual, visual CAC assessment and standard CACS, on non-ECG-gated CT. In a simple CAC assessment, we manually measured the length of the longest calcified lesions of coronary arteries (Figure 1). The presence of CAC was visually evaluated. When CAC was observed in a series of slices in a craniocaudal direction, the length of CAC was obtained as a sum of the slices. The length of CAC was independently analyzed by two blinded cardiologists on the right, left anterior descending, and left circumflex arteries and was assessed with the arbitrarily defined three thresholds for each coronary artery: 5, 10, and 15 mm. In this manual CAC assessment, the relationship between the number of coronary arteries with a length of CAC > 5, 10, or 15 mm and the presence of epicardial CAD on invasive angiography was evaluated. In the representative case in Figure 1, the patient had CAC only in the left circumflex artery with a length of 23.8 mm. Thus, this patient had one coronary artery with CAC > 5, 10, and 15 mm.

Standard CACS was also calculated using a semiautomatic analysis software SYNAPSE VINCENT (version 5, Fujifilm, Tokyo, Japan). In CACS calculation, CAC was defined as a lesion of >130 Hounsfield units (HU) with an area > 0.51 mm^2^. The software computed lesion-specific scores by multiplying the area of each calcification by the corresponding CT value (scored as 1 for values between 131 and 199 HU, 2 for 200–299 HU, 3 for 300–399 HU, and 4 for ≥400 HU), according to the Agatston method [10]. The Agatston score (i.e., standard CACS) is derived by integrating the product of the total plaque area and a cofactor based on the attenuation of the plaque calcium, in HU. The Agatston score represents a weighted sum of CAC, accounting for the total area and maximal attenuation of calcification, and is well-validated and widely used in clinical practice, thereby serving as a reference standard [8] CACS > 400 was defined as being significant in the present study [1,11].

### 2.3. Statistical Analysis

All statistical analyses were performed using EZR (Saitama Medical Center, Jichi Medical University, Saitama, Japan), which is a graphical user interface for R (The R Foundation for Statistical Computing, Vienna, Austria) [12]. Data are expressed as mean ± standard deviation, median [interquartile range], or frequency (percentage). Continuous variables were evaluated using Student’s *t*-test, and categorical variables were compared with Fisher’s exact test. Receiver operating characteristic (ROC) curve analysis was performed to evaluate the diagnostic ability of the number of coronary arteries with a length of CAC > 5, 10, or 15 mm and CACS for estimating epicardial CAD with area under the curve (AUC). The best cut-off value was established by finding the values that corresponded to the maximum average sensitivity and specificity. AUCs were compared using the Delong method. The inter-observer agreement of simple CAC assessment was evaluated with Cohen’s kappa coefficient with 95% confidence intervals. The kappa values of <0.20, 0.21–0.40, 0.41–0.60, 0.61–0.80, and 0.81–1.00 were considered slight, fair, moderate, substantial, and almost perfect, respectively [13]. A *p* value < 0.05 was considered statistically significant.

## 3. Results

Of the 100 patients, 49 (49.0%) had significant epicardial CAD on invasive coronary angiography. Baseline patient characteristics are listed in Table 1. Overall, the mean age was 70.5 ± 11.5 years, men accounted for 74.0%, and the mean body mass index was 23.3 ± 5.7 kg/m^2^. Overall, cardiovascular risk factors including hypertension, dyslipidemia, diabetes, and current smoking were frequent with a prevalence of 60.0%, 41.0%, 34.0%, and 29.0%, respectively. The leading indication of coronary angiography was heart failure (42.0%), followed by suspected angina (29.0%), preoperative testing (13.0%), brady arrhythmia (8.0%), peripheral artery disease (6.0%), and vasospastic angina (2.0%). When dividing patients into two groups, those with CAD were more likely to have cardiovascular risk factors such as hypertension (71.4% vs. 49.0%, *p* = 0.02), diabetes (57.1% vs. 25.5%, *p* = 0.001), and dyslipidemia (44.9% vs. 23.5%, *p* = 0.02) as compared to those without (Table 1). An estimated glomerular filtration rate (65.3 ± 21.5 vs. 59.4 ± 16.3 mL/min/1.73 m^2^, *p* = 0.13) and the rate of chronic kidney disease (39.2% vs. 46.9%) did not differ significantly between the two groups. Of the 49 patients with CAD, 40 (81.6%) subsequently underwent coronary revascularization and 9 (18.4%) were conservatively treated, respectively.

**Figure 2:**
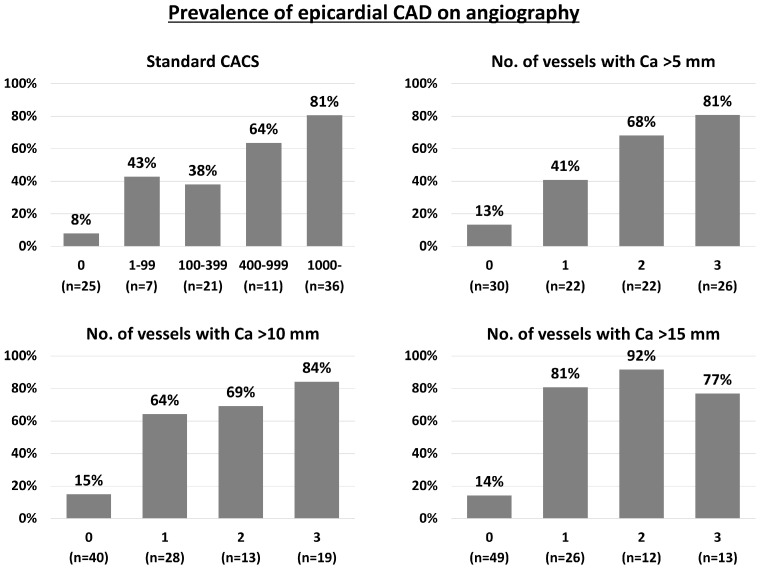
Prevalence of CAD on angiography based on coronary calcification assessment on non-ECG-gated CT. CACS, coronary artery calcification score; CAD, coronary artery disease; CT, computed tomography; ECG, electrocardiography.

**Figure 3:**
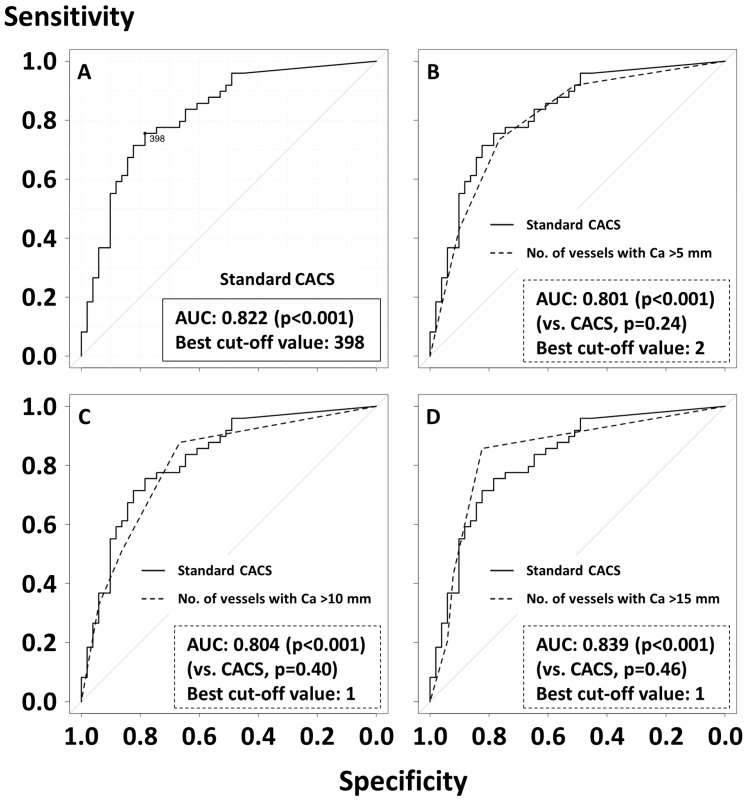
Receiver operating characteristic curve analysis for the presence of epicardial CAD. AUCs are compared between standard CACS (**A**) and the number of coronary arteries with the length of coronary calcium > 5 (**B**), 10 (**C**), or 15 (**D**) mm. AUC, area under the curve; CACS, coronary artery calcification score; CAD, coronary artery disease.

**Figure 4:**
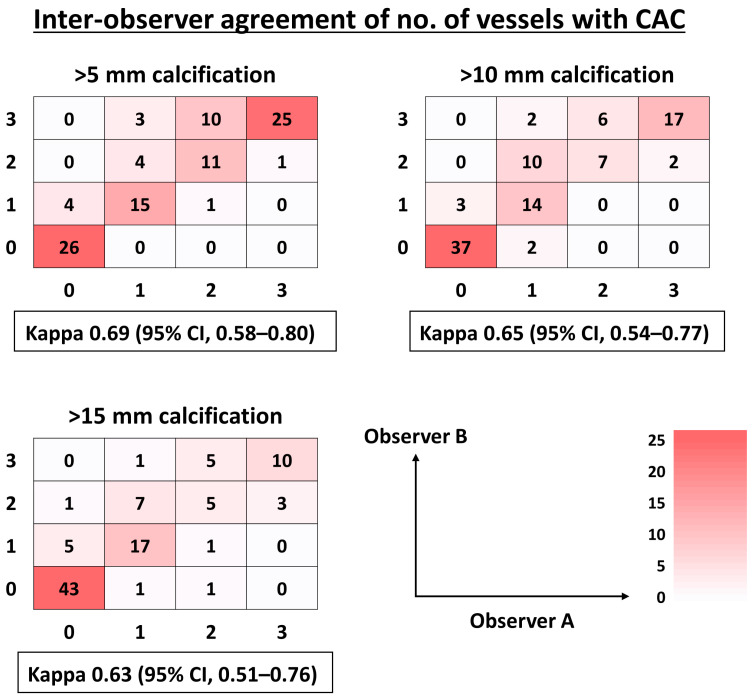
Inter-observer agreement of manual calcification length assessment. CAC, coronary artery calcification; CI, confidence interval.

Overall, 70 out of 100 (70.0%) patients had at least one calcified coronary lesion > 5 mm, and the median CACS was 346 [7, 1965]. Significant CAC (i.e., CACS > 400) was observed in 47.0%. In both manual CAC assessment and standard CACS, the increase in CAC burden was progressively associated with the presence of epicardial CAD on invasive coronary angiography (Figure 2). For instance, the prevalence of epicardial CAD on angiography was 13%, 41%, 68%, and 81% in patients with 0, 1, 2, and 3 vessels with a length of CAC > 5 mm. The ROC curve analysis showed that both manual CAC assessment and standard CACS were all predictive for CAD, with no statistically significant difference in AUCs between the two methods (Figure 3). The AUC of standard CACS was 0.822 with the best cut-off value of 398, while AUCs of the number of coronary arteries with the length of coronary calcium > 5, 10, and 15 mm were 0.801, 0.804, and 0.839. The best cut-off values for predicting the presence of epicardial CAD were 2, 1, and 1 for the number of vessels with CAC > 5, 10, and 15 mm, respectively (Figure 3). Sensitivity, specificity, positive predictive value (PPV), negative predictive value (NPV), and accuracy of simple CAC assessment and standard CACS > 400 were summarized in Table 2. Sensitivity, specificity, PPV, NPV, and accuracy of standard CACS > 400 were 73%, 78%, 77%, 75%, and 76%, and, for instance, those of two coronary arteries with the length of coronary calcium > 5 mm were 73%, 76%, 75%, 75%, and 75%, respectively. Sensitivity, specificity, PPV, NPV, and accuracy of one coronary artery with a length of coronary calcium > 15 mm were 86%, 82%, 82%, 86%, and 84%. Overall, the diagnostic ability of simple CAC assessment was similar to that of standard CACS (>400) with the cut-off values identified by the ROC curve analysis (Table 2). Figure 4 illustrates that inter-observer agreement of simple CAC assessment was substantial, with the kappa values ranging from 0.63 to 0.69.

## 4. Discussion

The present study demonstrated that manual CAC assessment on non-ECG-gated plain CT was feasible and had a similar diagnostic ability to standard CACS for estimating the presence of significant epicardial CAD on invasive angiography. The inter-observer variability of manual CAC assessment was acceptable. Because this diagnostic approach needs no dedicated techniques and equipment, it may be clinically relevant when evaluating the risks of ASCVD.

Vascular calcification, originally described as a bone-like artery wall, resembles bone mineralization [14]. There are two major types of vascular calcification, atherosclerosis-associated intimal calcification and diabetes and kidney disease-associated medial calcification. Calcium phosphate hydroxyapatite crystals deposit into the extracellular matrix, leading to the death of vascular smooth muscle cells and instability of plaques in intimal calcification and/or vessel stiffening and reduced compliance in medial calcification. In the process of atherosclerosis, arterial intima is calcified. Within atherosclerotic plaques, morphologies of intimal calcification mainly include two mechanisms: (1) micro or spotty early-stage calcifications and (2) macro or sheet-like late-stage calcifications [14]. The microcalcification progresses with released matrix vesicles surrounding the lipid plaques and provides inflammatory stimulus, while the macrocalcification has plaque-stabilizing properties. Within the macrocalcification lesions, macrophages can resolve the inflammation and stabilize the plaques [14]. In addition, recent investigations have provided detailed mechanistic insights into arterial calcification, particularly with significant roles of sirtuin 6 and indoleamine 2,3-dioxygenase 1 [15,16].

CAC is formed as a result of atherosclerotic plaques, and CACS on non-contrast-enhanced CT has been established as a screening test for epicardial CAD and a clinical examination to stratify cardiovascular risks [1,2,7,17]. Accurate risk estimation may provide better patient care, and it is well known that objective risk assessment rather than intuitive physician’s assessment is more useful in risk discrimination. The prospective MESA study, a population-based cohort including White, Black, Hispanic, or Chinese without known cardiovascular disease, showed that the addition of CACS to a risk-stratifying model based on traditional cardiovascular risk factors significantly improved the risk classification, particularly in those with higher risks [18]. Recent guidelines recommend CACS assessment in individuals with an intermediate cardiovascular risk [4,5,11], although a clinical benefit of risk-based preventive interventions has not been established [19]. While standard CACS is usually evaluated on ECG-gated CT to minimize motion artifacts [8], CACS assessment with no ECG-gating may be also useful in predicting cardiovascular outcomes [9]. Indeed, a meta-analysis demonstrated that the diagnostic agreement of CACS between CT scans with and without ECG-gating was almost perfect with a kappa value of 0.89 [9]. However, CACS calculation needs dedicated software, which may be a barrier when evaluating the calcification score. In this context, we assessed the diagnostic ability of manual CAC assessment on non-ECG-gated CT for epicardial CAD.

In 2016, the Society of Cardiovascular Computed Tomography and the Society of Thoracic Radiology guidelines for CACS indicated that CAC may be visually assessed and reported at all plain, non-gated chest CT examinations as none, mild, moderate, or severe [20], despite being subjective. A previous study showed the diagnostic potential of visually estimated Agatston score with the 6-level scale of 0, 1 to 9, 10 to 99, 100 to 300, 400 to 999, or ≥1000, although this approach was based on the requirement of active learning and educational feedback [21]. In the present study, we manually and semi-quantitatively evaluated calcification in coronary arteries. The ROC curve analysis indicated that the best cut-off value of CACS for CAD was 398 in this study, which was close to the established threshold of 400 [1,11]. In the individual patient data meta-analysis of the international COME-CCT Consortium, a total of 2452 patients from 76 studies who underwent both CACS assessment on CT and invasive coronary angiography were included [22]. The presence of significant obstructive (epicardial) CAD was defined as a diameter reduction of ≥50% on invasive angiography. In this international study, standard CACS > 400 reportedly had relatively high specificity and positive predictive value with an accuracy of around 70% for the presence of angiographically significant epicardial CAD, which may be in line with our results (i.e., 76%) [22]. In the COME-CCT Consortium study, the prevalence of significant obstructive CAD on invasive angiography in patients with standard CACS of 0, 1–99, 100–399, 400–999, and ≥1000 was 16.8%, 35.6%, 55.5%, 70.4%, and 78.5%, respectively [22]. In the present study, the prevalence in the same categories was 8%, 43%, 38%, 64%, and 81%, respectively. These findings suggest that our standard CACS assessment on non-ECG-gated CT may be acceptable. Agatston et al. originally reported the cut-off value of CACS of 300 in patients with significant epicardial CAD (percentage of diameter stenosis > 50%), while subsequent reports have indicated a CACS cut-off value of 400 for predicting significant myocardial ischemia and major adverse cardiovascular events [7]. Beyond standard CACS, we showed the feasibility of manually assessed CAC on non-ECG-gated plain CT in this study, in which the best cut-off values for predicting the presence of epicardial CAD were 2, 1, and 1 for the number of vessels with CAC > 5, 10, and 15 mm, respectively. The diagnostic accuracy of two vessels with CAC > 5 mm, one with CAC > 10 mm, and one with CAC > 15 mm was 75%, 77%, and 84%, respectively. Given the numerically higher accuracy and relatively higher sensitivity as compared to standard CACS, the presence of CAC > 10 and 15 mm in at least one coronary artery on non-ECG-gated chest CT may be clinically useful in identifying significant CAD, with no specific techniques and equipment needed. Our simple CAC assessment can be quickly performed offline and may be resource-saving. Although the randomized SCOT-HEART trial clearly demonstrated that the use of CT angiography on top of standard care in patients with stable chest pain significantly reduced the risk of death related to CAD and myocardial infarction at 5 years than standard care alone [23], whether CACS-guided therapeutic intervention improves clinical outcomes remains uncertain. In the US guidelines, statin treatment on the basis of ASCVD risk combined with CACS assessment is recommended [4]. In patients with a 10-year ASCVD risk ≥ 7.5% to <20%, the initiation of statin is not recommended when CACS is zero, but such treatment is indicated for those with CACS ≥ 100 [4]. The therapeutic potential of CACS-guided strategies deserves validation with randomized clinical trials.

Another point to note is the repeatability of the CACS assessment. Although CACS assessment is primarily indicated for the initial or baseline risk estimation, repeated CACS assessment may be clinically useful in evaluating the development of atherosclerosis and can be allowed in real-world practice because of the low invasiveness, low inter-exam variability, and low cost [8]. In the MESA study, CAC on CT was repeatedly evaluated in a total of 3382 participants free of clinical cardiovascular disease [24]. In this multiethnic study, the median change in CACS per year was 28.9, and the greater progression of CACS was progressively associated with a greater risk of coronary heart disease [24]. Although the clinical usefulness of serial evaluation of CACS has not been established yet, this diagnostic strategy may contribute the better ASCVD risk stratification. Furthermore, the progression of CACS may be related to the efficacy of current management including lifestyle modification and medications, thereby prompting the reassessment of whether more aggressive therapeutic strategies are needed. Indeed, the Society of Cardiovascular Computed Tomography guidelines recommend repeat CACS at 5 years in patients with an initial CACS of 0 and at 3 to 5 years in those with CACS > 0 [25]. Even with chest CT images that were unintentionally scanned for CAC assessment, repeated CACS can be easily evaluated using our simple CAC assessment with no dedicated software. Cautions may be warranted in evaluating CACS in patients who are receiving statins, because the score might be falsely assessed despite the lower ASCVD risk, possibly owing to the calcification of previously soft (lipid) coronary plaques

The present study had some limitations. This was a retrospective study conducted in a retrospective, single-center manner. Despite the acceptable inter-observer agreement, further prospective, large-scale studies are warranted to confirm our results. In the present study, significant epicardial CAD was found in 49.0%, while the prevalence in previous studies of non-invasive stress testing often ranged from 7% to 29% [1,26,27]. Because all patients in this study underwent invasive angiography with clinical indications, the prevalence of CAD may have been higher than that in previous reports. In a recent study (n = 2452) in which all participants underwent coronary angiography, 44.9% had obstructive CAD, defined as coronary diameter stenosis > 50% on angiography [22]. Although ischemic and functional testing was not necessarily mandated [27], most patients with obstructive CAD underwent coronary revascularization in a real-world setting in the present study [28,29,30,31]. In addition, a slice interval was 1.25 mm in this study, which may be thinner than that in previous studies [9]. Whether the present study results can be replicated using CT images with other slice intervals (e.g., 2.5 and 5.0 mm) is unclear. Although the thresholds of 5, 10, and 15 mm for evaluating the length of CAC in each coronary artery in the present study may be reasonable, these cut-off values were arbitrarily defined.

## 5. Conclusions

Manually evaluated coronary calcium length on non-ECG-gated, non-contrast enhanced chest CT was predictive for the presence of significant epicardial CAD on invasive coronary angiography with similar diagnostic ability to standard CACS. Because our CAC assessment does not need dedicated equipment and can be analyzed offline, this simple approach may be useful in estimating CAD risks.

## Figures and Tables

**Table 1:** Baseline characteristics.

Variable	All (*n* = 100)	Epicardial CAD (>50%) on CAG		*p* Value
		No (*n* = 51)	Yes (*n* = 49)	
Age (years)	70.5 ± 11.5	68.7 ± 13.5	72.5 ± 8.7	0.10
Men	74 (74.0%)	34 (66.7%)	40 (81.6%)	0.88
BMI (kg/m^2^)	23.3 ± 5.7	24.4 ± 6.9	22.3 ± 4.2	0.17
Hypertension	60 (60.0%)	25 (49.0%)	35 (71.4%)	0.02
Diabetes	41 (41.0%)	13 (25.5%)	28 (57.1%)	0.001
Dyslipidemia	34 (34.0%)	12 (23.5%)	22 (44.9%)	0.02
Current smoker	29 (29.0%)	17 (33.3%)	12 (24.5%)	0.32
eGFR (mL/min/1.73 m^2^)	62.0 ± 19.3	65.3 ± 21.5	59.4 ± 16.3	0.13
Chronic kidney disease	43 (43.0%)	20 (39.2%)	23 (46.9%)	0.44
Indication of CAG				
Heart failure	42 (42.0%)	27 (52.9%)	15 (30.6%)	0.02
Suspected angina	29 (29.0%)	9 (17.6%)	20 (40.8%)	0.01
Preoperative testing	13 (13.0%)	5 (9.8%)	8 (16.3%)	0.33
Brady arrhythmia	8 (8.0%)	6 (11.8%)	2 (4.1%)	0.15
PAD	6 (6.0%)	2 (3.9%)	4 (8.2%)	0.37
Vasospastic angina	2 (2.0%)	2 (3.9%)	0 (0%)	0.16

BMI, body mass index; CAD, coronary artery disease; CAG, coronary angiography; eGFR, estimated glomerular filtration rate; PAD, peripheral artery disease.

**Table 2:** Diagnostic ability of cut-off values for predicting the presence of epicardial CAD.

	Sensitivity (%)	Specificity (%)	PPV (%)	NPV (%)	Accuracy (%)
Standard CACS > 400	73	78	77	75	76
No. of vessels with Ca > 5 mm					
1	92	51	64	87	71
2	73	76	75	75	75
3	43	90	81	62	67
No. of vessels with Ca > 10 mm					
1	88	67	72	85	77
2	51	86	78	65	69
3	33	94	84	59	64
No. of vessels with Ca > 15 mm					
1	86	82	82	86	84
2	43	92	84	63	68
3	20	94	77	55	58

Ca, calcification; CACS, coronary artery calcification score; CAD, coronary artery disease; NPV, negative predictive value; PPV, positive predictive value.

## Data Availability

The data of this study are available upon reasonable request.
